# Multiple triggering mechanisms of myocardial fibrosis: Comparison and integration in different disease contexts

**DOI:** 10.1016/j.isci.2026.116734

**Published:** 2026-07-14

**Authors:** Bingru Zhao, Xiaoya Chen, Shuhui Feng, Junjie Pang, Hongyuan Liu, Haoyue Yang, Wen Li, Huanzhen Chen

**Affiliations:** 1 First Clinical Medical College, Shanxi Medical University, Taiyuan, China; 2 Department of Cardiology, First Hospital of Shanxi Medical University, Taiyuan, China; 3 University of Chinese Academy of Sciences, Beijing, China

**Keywords:** myocardial fibrosis, cardiovascular diseases, mechanisms

## Abstract

Myocardial fibrosis (MF) is a pathophysiological process characterized by the excessive deposition of collagen fibers in the myocardium. Numerous studies are now dedicated to clarifying the genetic, cellular, and molecular mechanisms that drive these pathological alterations. Furthermore, substantial evidence suggests that diffuse fibrosis causes irreversible damage to the heart. Myocardial fibrosis and cardiovascular disease are interrelated, influencing each other significantly. However, the existing evidence linking myocardial fibrosis to common diseases is still insufficient. In this review, we summarized the common mechanisms underlying myocardial fibrosis and their associations with prevalent cardiovascular diseases (e.g., myocardial infarction, heart failure, hypertrophic cardiomyopathy, dilated cardiomyopathy), and emphasized the heterogeneous presentations of myocardial fibrosis across different diseases to promote the future realization of personalized treatment for fibrosis-related cardiovascular diseases.

## Graphical abstract

**Figure:**
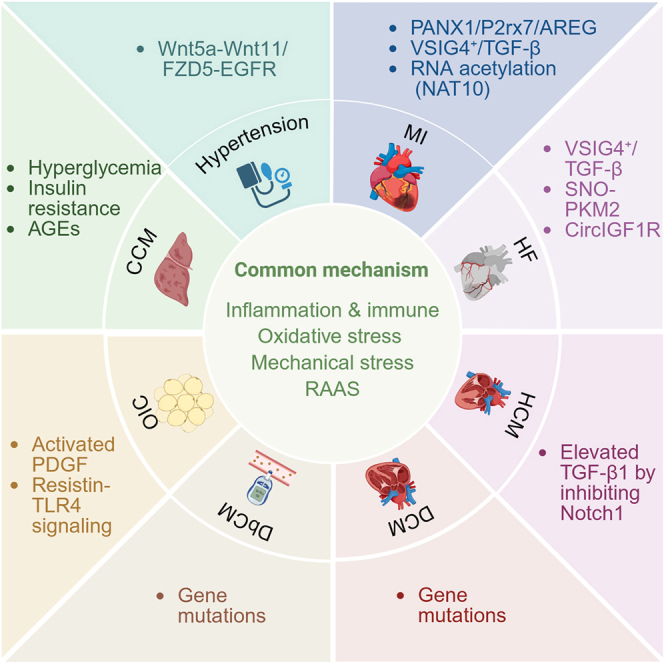

## Teaser

Health sciences; Medicine; Medical specialty; Internal medicine; Cardiovascular medicine

## Introduction

Myocardial fibrosis (MF), characterized by the excessive deposition of extracellular matrix (ECM) proteins such as collagens, leads to the destruction of myocardial structure, increased tissue stiffness, and impaired cardiac contractility. It is a fundamental mechanism driving the progression from diverse cardiac injuries to end-stage heart failure (HF). The initiating triggers and early pathogenic mechanisms of MF vary significantly across different disease contexts.^1^ For instance, the post-infarction milieu is dominated by acute inflammation and cardiomyocyte death,^1^ while pressure overload in hypertension and HF with preserved ejection fraction (HFpEF) engages potent mechanosensitive and neurohormonal pathways. In contrast, dilated cardiomyopathy (DCM) often involves genetic mutations that directly perturb cardiomyocyte integrity and signaling. While the endpoint of MF may be similar, the disease-specific initiating triggers and predominant molecular pathways involved are highly heterogeneous.^2^ This underlying heterogeneity poses a fundamental therapeutic challenge. Current therapeutic approaches, although effective in managing hemodynamics and neurohormonal activation, largely fail to directly and adequately halt or reverse the fibrotic process.

Despite the diverse initiating triggers across different cardiac diseases, these upstream signals ultimately converge on a common set of downstream fibrotic signaling cascades—most notably the TGF-β/Smad, mitogen-activated protein kinase (MAPK), and PI3K/Akt pathways—that serve as central executioners of fibrosis. Moreover, recent discoveries have unveiled a wealth of novel mechanisms, including purinergic signaling via pannexin1 (PANX1) channels,^3^ macrophage phenotype regulation,^4^ RNA epitranscriptomics,^5^ and fibroblast senescence.^6^ Although these investigations are still confined to particular disease models, they can act as upstream signals of the core hub or direct regulatory factors affecting the core pathway.

Therefore, a systematic comparison and integration of how different upstream insults activate these convergent fibrotic cascades is critical. This review aims to dissect the multifaceted mechanisms of MF across four major cardiac conditions: myocardial infarction (MI), HF, cardiomyopathy, and hypertension. The article also provides novel insights for formulating precise and personalized anti-fibrotic treatment strategies.

## Shared pathogenic mechanisms underlying cardiac fibrosis

**Figure 1:**
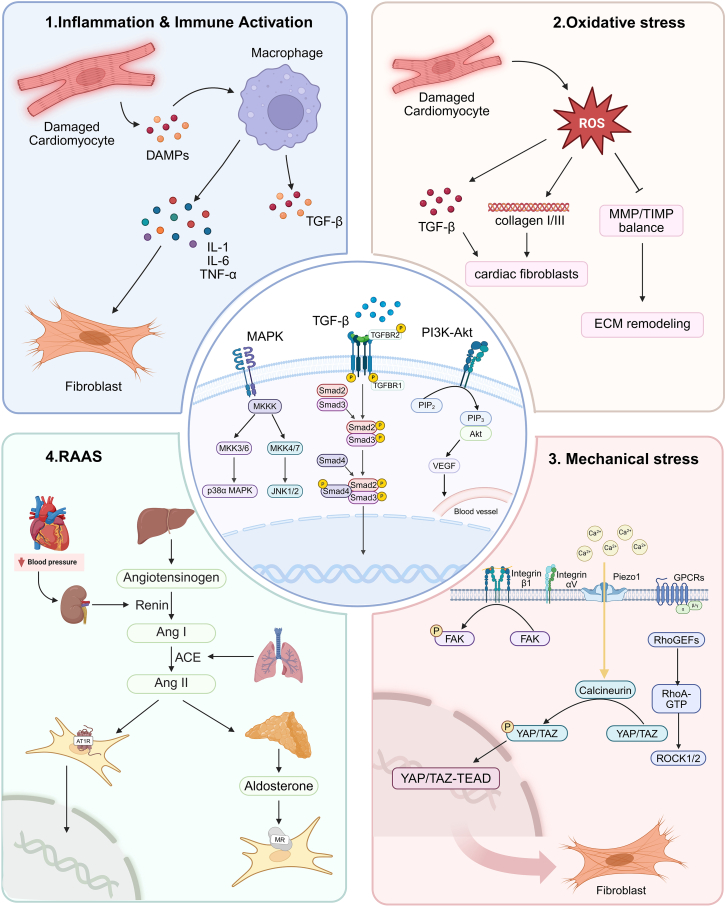
The core triggering mechanisms and shared signaling networks regulating myocardial fibrosis Myocardial fibrosis (MF) is driven by a complex interplay of systemic and local triggers that converge on a set of core pathogenic pathways. (1) Inflammation and immune activation: Tissue injury or cardiomyocyte death triggers the release of danger-associated molecular patterns (DAMPs), which activate resident and recruited macrophages. This inflammatory response culminates in the secretion of pro-fibrotic cytokines, establishing a profibrotic microenvironment. (2) Oxidative stress: Damaged cardiomyocytes and metabolic enzymes, such as NADPH oxidase 4 (NOX4), generate reactive oxygen species (ROS). ROS directly activate cardiac fibroblasts and amplify TGF-β signaling. (3) Mechanical stress: Pathological mechanical stretch is sensed by mechanoreceptors, including Integrins (β1 and αV), the transient receptor potential (TRP) cationic channels, Piezo1, and G protein-coupled receptors (GPCRs). These signals are transduced via Focal Adhesion Kinase (FAK) and the RhoA/ROCK cascade to promote fibroblast-to-myofibroblast trans differentiation. YAP/TAZ activation potentiates the TGF-β signaling pathway and directly stimulates fibroblast proliferation. (4) RAAS Activation: The RAAS cascade generates Ang II and aldosterone. Ang II acts through the AT1R to trigger canonical TGF-β signaling, while aldosterone engages mineralocorticoid receptors (MRs) to stimulate fibrotic programs via MAPK signaling. Convergent Downstream Signaling: These diverse triggers converge on the canonical TGF-β/Smad axis and non-canonical pathways, including the MAPK cascades and the PI3K/Akt pathway. Together, these pathways orchestrate extracellular matrix (ECM) deposition and the sustained activation of myofibroblasts.

Although the pathological features of cardiac fibrosis—such as its severity, localization, and clinical impact—vary depending on the underlying disease, a set of core initiating mechanisms, including inflammation, renin-angiotensin-aldosterone system (RAAS) activation, mechanical stress, and oxidative stress, is universally shared (Figure 1).

### Inflammation and immune activation

Cardiomyocyte damage or death triggers the release of danger signals known as danger-associated molecular patterns (DAMPs),^7^ which activate the innate immune system, particularly macrophages.^8^ This activation results in the release of various pro-inflammatory and profibrotic cytokines, including IL-1, IL-6, TNF-α, and TGF-β.^9^ Collectively, these factors create a profibrotic microenvironment where cytokines such as IL-17, TNF, and IL-1β can directly activate fibroblasts,^10^ thereby promoting their ECM production.^9^ TGF-β plays a crucial role in the trans-differentiation of cardiac fibroblasts and the deposition of ECM.^7^ In injured cardiomyocytes, TGF-β regulates the function of fibroblasts and immune cells through both the Smad and non-Smad pathways, such as MAPK and PI3K/Akt.^8^

### Oxidative stress

Concomitantly, injured cardiomyocytes generate reactive oxygen species (ROS), which further contribute to the pathological process. In cardiac tissue, the presence of ROS promotes the upregulation of TGF-β1, α-smooth muscle actin (α-SMA), and collagen I/III, thereby activating cardiac fibroblasts.^11^ TGF-β is pivotal in the process of fibrosis. Simultaneously, ROS disrupt the equilibrium between matrix metalloproteinases (MMPs) and their inhibitors, leading to fibrosis and alterations in matrix structure.^11^ Additionally, ROS can trigger apoptosis via caspase activation and induce oxidative damage to ECM proteins, ultimately hindering the resolution of inflammation and fibrosis.^11^ Moreover, some metabolic enzymes, such as nicotinamide adenine dinucleotide phosphate (NADPH) oxidases (Noxs), also generate ROS. NOX4, a member of the Noxs family, mediates TGF-β-induced fibrosis through the amplification of Smad2/3 signaling.^12^

### Mechanical stress

Mechanical stress can directly activate cardiac fibroblasts. Under conditions of sustained mechanical stress, cardiac fibroblasts detect and transduce biomechanical signals through mechanosensitive receptors, such as integrins, G protein-coupled receptors (GPCRs), and growth factor receptors,^13^ which in turn activate downstream pathways that promote matrix fibrosis.

Integrins are classical transmembrane receptors in terms of mechanotransduction. They mediate cell-ECM interactions and transduce mechanical, matricellular, and growth factor signals.^13^ Integrins with different subunits play different roles in MF. Integrin β1 acts as the predominant receptor for sensing pathological mechanical stretch in cardiac fibroblasts. It mediates the synthesis of new collagen in the pressure-overloaded heart and directly activates fibroblasts under mechanical stress.^13^ Integrin αV (CD51) predominantly functions as a co-receptor that binds and activates latent TGF-β, thereby amplifying the canonical Smad-dependent pro-fibrotic cascade.^14^ Focal adhesion kinase (FAK) is an important mediator of integrin-mediated transduction. Given that integrin cytoplasmic domains possess no endogenous catalytic activity, signal propagation relies on adaptor kinases such as FAK.^15^ Under sustained mechanical load, clustered Integrin β1 recruits and activates FAK via phosphorylation.^15^ Notably, FAK is related to collagen deposition under mechanical stimulation.^16^

Furthermore, TGF-β activates members of the transient receptor potential (TRP) family of cationic channels, which serve essential functions in fibroblast activation and the pathogenesis of MF. Specifically, TRPM7,^17^ TRPC6,^18^ and TRPV4^19^ have each been implicated in TGF-β1 driven fibrogenesis. Among these, TRPC6 mediates hypertrophic responses in cardiac myocytes^18^ and increases pressure-induced myofibroblast differentiation.^13^ TRPM7^17^ and TRPV4^19^ play a crucial role in TGF-β1-induced fibrogenesis. Similarly, the mechanosensitive ion channel Piezo1 is critically involved in fibrotic remodeling of the injured myocardium. Elevated mechanical stress following cardiac injury upregulates Piezo1 expression in cardiac fibroblasts, which in turn triggers calcium influx and initiates fibroblast-to-myofibroblast transdifferentiation.^20^

Activation of GPCRs and integrins promotes the recruitment of Rho guanine nucleotide exchange factors (RhoGEFs), which stimulate the small GTPase RhoA. Activated RhoA subsequently transduces signals through its primary downstream effectors, the Rho associated coiled coil containing kinases ROCK1 and ROCK2.^21^ Both ROCK isoforms have been implicated in fibroblast activation, with distinct contributions reported for ROCK1 and ROCK2.^22^^,^^23^ Notably, the RhoA/ROCK signaling cascade serves as a key downstream mediator of TGF-β signaling in the context of fibrosis.^24^ There are also studies indicating that the homologous transcriptional coactivators yes associated protein (YAP) and transcriptional co activator with PDZ binding motif (TAZ) serve as pivotal mediators of cellular responses to mechanical stress. In the context of fibrotic remodeling, YAP/TAZ activation potentiates the TGF-β signaling pathway and directly stimulates fibroblast proliferation.^25^ Moreover, YAP/TAZ signaling modulates cardiac fibrosis not only through direct pro-fibrotic transcriptional programs but also regulates cardiac fibrosis by suppressing the inflammatory response.^26^

### Activation of the renin angiotensin aldosterone system

The RAAS is a convergent neurohormonal pathway shared by almost all major cardiac diseases. The biochemical cascade of the RAAS proceeds as follows: angiotensinogen, produced by the liver, is first cleaved by renin to form angiotensin I (Ang I). Ang I is subsequently hydrolyzed by angiotensin-converting enzyme (ACE) to generate angiotensin II (Ang II). Ang II and its downstream effector aldosterone constitute the major effector molecules of the RAAS. Disease-related stress triggers the activation of cardiomyocytes, immune cells, and cardiac fibroblasts, leading to upregulation of local renin and ACE expression. This promotes the intracardiac generation of Ang II.^27^ Ang II plays a vital role in activating cardiac fibroblasts.^21^ First, Ang II binds to the angiotensin II type 1 receptor (AT1R), activating the canonical TGF-β signaling pathway.^28^ Beyond its direct effects, Ang II stimulates aldosterone secretion from the adrenal glands. Aldosterone exerts additional pro-fibrotic effects by binding to mineralocorticoid receptors (MRs) on cardiac fibroblasts and cardiomyocytes to stimulate cardiac fibroblasts via MAPK signaling.^29^

### Core downstream signaling pathways mediating myocardial fibrosis

The TGF-β/Smad axis as the central hub. Upon TGF-β binding to its receptor, TGF-β receptor 2 (TGFBR2), the phosphorylation of TGFBR2 leads to the activation of TGF-β receptor 1 (TGFBR1).^30^ Subsequently, TGFBR1 activates downstream Smad proteins (Smad2 and Smad3) through phosphorylation, ultimately forming a heterocomplex. This complex performs specific biological functions and transmits TGF-β signaling within the nucleus. Downstream, Smad3 activates fibroblast synthesis of ECM proteins and promotes the formation of myofibroblasts.^31^ In addition to Smad signaling, TGF-β activates non-Smad routes such as MAPK, which cooperate with or amplify the fibrotic response. The intracellular MAPK signaling pathway responds to injury stress and promotes the expression of TGF-β. The MAPK signaling cascades consist of three key components: MAPK kinase (MKKK), MAPK kinase (MKK), and the terminal MAPK. Members of the MAPK family include the c-Jun N-terminal kinase (JNK) cascade, the p38 MAPK cascade, and the extracellular signal-regulated kinase 5 (ERK5) cascade, with p38 MAPK and JNK being particularly associated with cardiovascular diseases.^32^ The activation of the MKKK-MKK3/6-p38α MAPK and ASK1-MKK4/7-JNK1/2 cascades promotes pathological cardiac remodeling, including fibrosis. Beyond their direct cytosolic effects, these kinases can also phosphorylate the linker region of Smad2/3, thereby fine-tuning the transcriptional output of the canonical TGF-β/Smad axis.

Beyond these kinase cascades, metabolic reprogramming mediated by the PI3K/Akt pathway provides an additional layer of fibrotic support. The activation of the PI3K/Akt pathway initiates a biochemical cascade in which phosphatidylinositol 4,5-bisphosphate (PIP2) is transformed into phosphatidylinositol 3,4,5-trisphosphate (PIP3) through the action of PI3K. The produced PIP3 interacts with the pleckstrin homology domain of Akt, leading to a significant conformational change in Akt. As a result of this conformational change, Akt activates various downstream effector molecules, one of which is the vascular endothelial growth factor (VEGF), facilitating the formation of new capillaries. These newly formed capillaries provide the essential energy required for the transformation of fibroblasts into myofibroblasts.^33^

## Myocardial infarction and myocardial fibrosis

MI refers to the death of myocardial cells caused by sustained ischemia of the heart. The development of MF after MI is associated with an increased risk of major adverse cardiovascular events (MACEs), such as arrhythmias and sudden cardiac death.^3^ Therefore, elucidating the pathological mechanisms is essential for developing effective therapies. Following MI, the death of cardiomyocytes triggers an immune and inflammatory response. This inflammatory environment significantly activates canonical pro-fibrotic pathways, particularly the TGF-β/Smad axis,^34^ as well as the MAPK^32^ and PI3K pathways.^33^ This activation leads to fibroblast activation, myofibroblast differentiation, and ECM deposition.

Recent studies have identified novel mechanisms. After MI, dead myocardial cells release a large amount of ATP. Deng et al.^35^ discovered a signaling pathway related to ATP. They indicated that PANX1 orchestrates post-MI cardiac fibrosis through the PANX1/P2rx7/amphiregulin (AREG) signaling axis. PANX1 is a plasma membrane channel protein that plays a critical role in the execution of apoptosis.^35^ AREG, an epithelial growth factor, modulates inflammatory responses, tissue repair, and fibro genic processes through the activation of the epidermal growth factor receptor (EGFR).^36^ Mechanistically, opening of PANX1 channels in damaged cardiomyocytes mediates extracellular ATP release. This ATP recruits macrophages through purinergic receptor P2X7 (P2rx7) recognition, inducing AREG expression and ultimately exacerbating cardiac fibrosis in post-MI.^37^ This is one of the most direct pathways through which myocardial cell death directly drives fibrosis, converting injury signals into AREG. Notably, Clochard et al. demonstrated that Panx1 is expressed in human cardiac fibroblasts through an *in vitro* model of TGF-β-induced activation and *trans*-differentiation of these cells.^38^ Theoretically, circulating PANX1 or ATP could serve as early indicators of cardiomyocyte injury that precedes established fibrosis. However, to date, no clinical studies have evaluated the diagnostic or prognostic value of plasma PANX1 or ATP in patients with post-MI. A recent study has shown that the P2X7-Receptor antagonist A740003 exhibits potential protective effects of fibrotic changes after acute myocardial infarction (AMI).^39^

In addition to the role of myocardial cells in the fibrosis process after MI, other cells such as macrophages also play important roles. Macrophages play a crucial role in the regulation of inflammatory processes and the repair of tissue after MI. V-set and immunoglobulin domain-containing 4 (VSIG4), a complement receptor of the immunoglobulin superfamily, is expressed exclusively in a subset of tissue-resident macrophages.^40^ Research by Wang et al. indicated an increase in VSIG4^+^ macrophages after MI. VSIG4 facilitates scar formation in the wake of AMI while also boosting the levels of TGF-β1 and IL-10. TGF-β aids in the formation of myofibroblasts through the TGF-β/Smad signaling pathway. These results suggest a promising new target for immunomodulatory therapy aimed at treating Myo fibrosis after MI.^41^ Recent evidence from patients with HF has further substantiated the role of VSIG4 in cardiac fibrosis.^42^^,^^43^ In the future, targeting macrophage phenotypes represents a potential immunomodulatory strategy against fibrosis.

Furthermore, with the development of epigenetics, the relationship between post-MI fibrosis and it has gradually been discovered. Recently, it has been discovered that acetylation at the RNA level is associated with MF after MI. In their research, Li et al.^44^ provided compelling evidence that following MI, there is a notable upregulation of N-acetyltransferase 10 (NAT10) expression. NAT10 is crucial for catalyzing the acetylation of RNA, including BCL-XL mRNA. This study also reveals that NAT10 plays a significant role in regulating the proliferation and differentiation of cardiac fibroblasts into myofibroblasts. This process is mediated through the ac4C acetylation of BCL-XL mRNA, ultimately contributing to the development of MF in the aftermath of MI. Notably, NAT10 expression and RNA ac4C levels are elevated in both *in vitro* and *in vivo* cardiac remodeling models, as well as in human patients with cardiac hypertrophy.^45^ The presence of ac4C-modified RNAs in plasma represents an unexplored frontier. The development of sensitive assays to detect NAT10 or ac4C-modified RNA expression could open a new window for epigenetic diagnostics in cardiac fibrosis. Given that NAT10 inhibitors (such as Remodeling) have demonstrated efficacy in improving cardiac hypertrophy and vascular remodeling, targeting NAT10 can not only effectively block the fibrotic process by disrupting the TGF-β/TGFBR1 signaling axis, but also offers the advantage of avoiding broad immunosuppression compared to traditional TGF-β inhibitors. Combined with nano delivery technologies, this approach holds promise as a safe and clinically translatable novel strategy for anti-fibrotic treatment after MI.^46^

**Figure 2:**
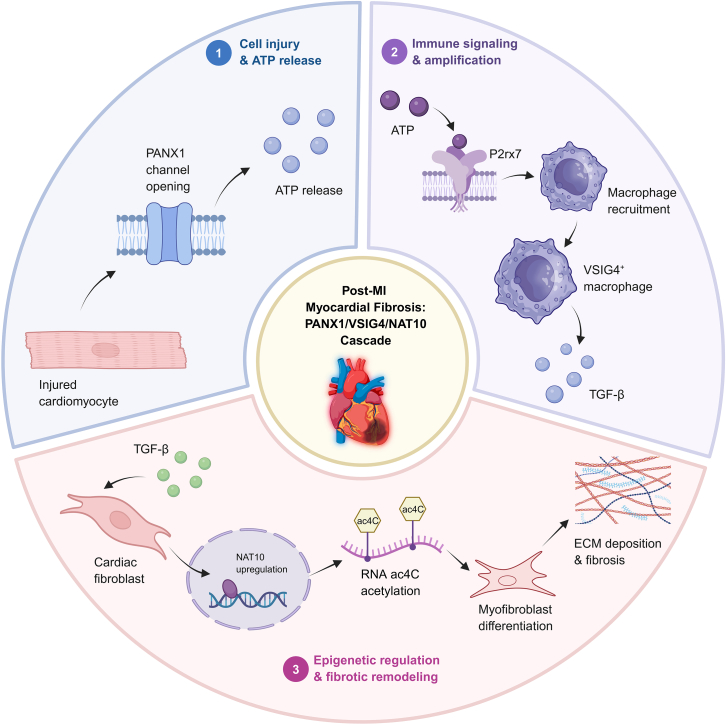
Diagram of the mechanisms of myocardial fibrosis induced by different types of cardiomyopathy Schematic overview of emerging signaling pathways driving myocardial fibrosis following MI. Damaged cardiomyocytes release ATP through opened PANX1 channels, which recruits macrophages via purinergic receptor P2X7 (P2rx7) recognition, inducing amphiregulin (AREG) expression that activates EGFR signaling to promote cardiac fibrosis. VSIG4, expressed on a subset of tissue-resident macrophages, is upregulated post-MI and further amplifies TGF-β1 and IL-10 secretion, driving myofibroblast differentiation through the canonical TGF-β/Smad pathway. Additionally, upregulated N-acetyltransferase 10 (NAT10) catalyzes ac4C acetylation of BCL-XL mRNA, promoting fibroblast-to-myofibroblast transition via epigenetic remodeling. These pathways form a functional cascade that collectively drives the development of cardiac fibrosis following myocardial infarction.

The existing findings indicate that PANX1, VSIG4, and NAT10 may be functionally linked. Following cardiac injury, a large amount of ATP is released through PANX1 channels on damaged cells and subsequently recruits macrophages via P2rx7.^37^ These macrophages mediate post-infarction fibrosis by secreting TGF-β-related factors. At the same time, VSIG4, which is highly expressed on a subset of macrophages, further amplifies TGF-β secretion.^41^ TGF-β then upregulates NAT10 expression in fibroblasts and initiates RNA ac4C acetylation-mediated fibrotic remodeling.^44^ Thus, these molecules form a functional cascade that links injury signals, immune response, and epigenetics (Figure 2).

In addition to the signaling pathways mentioned above, there are other emerging perspectives. For instance, inhibiting the mTORC1/S6K/eIF-4B signaling pathway post-MI mitigates cardiac fibrosis.^47^ Furthermore, the premature aging of key fibroblast cells can exacerbate MF following MI.^48^

## Heart failure and myocardial fibrosis

While the acute injury phase of MI initiates fibrotic repair, the progression to chronic HF is characterized by persistent metabolic and structural alterations within the fibroblast compartment. HF is a prevalent, serious, and fatal. MF is a common characteristic in HF. From the perspective of disease progression, MF can significantly influence the trajectory of HF. Therefore, it is crucial to investigate the relationship between MF and HF.

Among the shared mechanisms, oxidative stress and mechanical stress are particularly prominent in HF. A substantial body of evidence indicates that mitochondrial dysfunction and the increased production of ROS are prominent features following HF.^49^ In cardiac myocytes, the mitochondrial electron transport chain is a major ROS source.^49^ After HF, mitochondrial ROS production escalates, leading to damage of mitochondrial DNA (mtDNA). Damaged mitochondria further exacerbate ROS production under oxidative stress, creating a vicious cycle of ROS generation.

Furthermore, the hemodynamic load on the heart is primarily categorized into two types: pressure overload and volume overload. HFpEF is predominantly characterized by pressure overload, whereas HF with reduced ejection fraction (HFrEF) is primarily associated with volume overload. Pressure overload primarily activates mechanotransduction pathways that converge on TGF-β/Smad signaling,^50^ promoting fibroblast activation and ECM deposition. In contrast, volume overload impairs collagen degradation, disrupting ECM homeostasis and facilitating adverse remodeling.^51^

Beyond the classical pathways discussed above, recent transcriptomic and proteomic studies have implicated VSIG4 in human HF. Analysis of single-cell RNA sequencing data from human failing hearts reveals that VSIG4 is expressed in monocyte and macrophage subsets within the cardiac tissue. Furthermore, communication analysis among cell subtypes indicates frequent interactions between VSIG4^+^ macrophages and fibroblasts, providing transcriptomic evidence for a potential role of the VSIG4 pathway in human cardiac fibrosis.^43^ Also, complementing the single-cell transcriptomic findings, the serum level of circulating VSIG4 protein is significantly elevated in patients with HF. Moreover, serum VSIG4 concentrations positively correlate with C-reactive protein (CRP), suggesting a relationship between VSIG4 pathway activation and systemic inflammatory burden.^42^ This research further indicates that VSIG4 may regulate MF through immune and inflammatory responses.

Emerging evidence indicates that the metabolic alterations in cardiac fibroblasts play a crucial role in MF associated with HF. Two recent studies exemplify this emerging mechanism. Nitric oxide-mediated nitrosative stress causes injury to cardiac tissue, and excessive nitric oxide can trigger S-nitrosylation (SNO) of specific cysteine thiols. Luo et al. indicated that the SNO of pyruvate kinase M2 (PKM2) at cysteines 49 and 326 is increased in the heart tissue of patients with HF. Mechanistically, they demonstrated that SNO-PKM2 enhances the activation of cardiac fibroblasts by promoting excessive mitochondrial fission and mitochondrial dysfunction.^52^ Additionally, Schmidt et al. identified that circIGF1R exhibited dysregulation specifically in cardiac fibroblasts through deep circular RNA (circRNA) sequencing of cardiac tissue from patients with HF. Mechanistically, circIGF1R regulates fibroblast proliferation by inhibiting glycolysis. Their findings show that the overexpression of circIGF1R has anti-fibrotic effects in cardiac fibroblasts derived from patients with HF.^53^

## Cardiomyopathy and myocardial fibrosis

Cardiomyopathy is a group of heterogeneous diseases characterized by abnormal cardiac morphology and function, which can be caused by either genetic factors or metabolic disorders. MF is a shared core pathophysiological alteration and key progression driver in multiple myocardial diseases, serving as the common pathological basis and final common pathway for the development and progression of most cardiomyopathies.^21^ In genetic cardiomyopathies, mutations in genes encoding cardiac sarcomeric proteins are central pathogenic mechanisms, with hypertrophic cardiomyopathy (HCM) and DCM being the most representative and common types of inherited cardiomyopathies associated with sarcomeropathies.^54^ In contrast to inherited cardiomyopathies, cardiomyopathy associated with metabolic syndrome is distinct, with major risk factors including obesity, insulin resistance, dyslipidemia, and diabetes. Currently, diabetic cardiomyopathy (DbCM) and obesity-induced cardiomyopathy (OIC) are the most extensively studied subtypes.^55^ Additionally, cirrhotic cardiomyopathy (CCM) represents an important subtype of metabolic-related cardiomyopathy, which arises from hepatic insufficiency in liver cirrhosis and is closely linked to systemic metabolic defects caused by hepatic dysfunction.^56^

A substantial body of evidence suggests that metabolism-related cardiomyopathy and genetically associated cardiomyopathy are not independent of each other; rather, they exhibit extensive overlap in etiology, mechanisms, and phenotypes. The genetic background of an individual can influence the susceptibility to metabolic stress.^57^ Conversely, metabolic disturbances can act as significant environmental factors, accelerating the myocardial remodeling process in inherited cardiomyopathies.^58^ In HCM, a combined proteomic and metabolomic study revealed that myocardial tissues from patients with HCM exhibit universal characteristics of enhanced glucose and glycogen metabolism, downregulated fatty acid oxidation, and reduced lipid storage. This alteration in energy metabolism may exacerbate MF by generating oxidative stress or metabolic intermediates that activate cardiac fibroblasts.^59^

**Figure 3:**
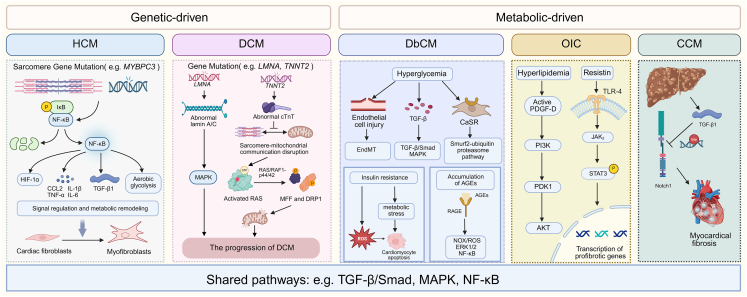
Diagram of the mechanisms of myocardial fibrosis induced by different types of cardiomyopathy Myocardial fibrosis (MF) serves as a convergent pathological outcome for diverse cardiomyopathies, categorized into genetic-driven and metabolic-driven etiologies. While initial triggers vary, they orchestrate a transition of cardiac fibroblasts into activated myofibroblasts through highly interconnected signaling networks. (1) Genetic-driven cardiomyopathies (HCM and DCM): Hypertrophic Cardiomyopathy (HCM): Sarcomere mutations (e.g., *MYBPC3*) trigger the NF-κB signaling cascade, promoting the release of pro-inflammatory cytokines (CCL2, IL-1β, IL-6, and TNF-α). This inflammatory milieu upregulates TGF-β1 and HIF-1α, driving aerobic glycolysis and metabolic remodeling to facilitate myofibroblast transformation. Dilated cardiomyopathy (DCM): Genetic variants such as *LMNA* mutations activate the MAPK (ERK/JNK) pathway. Additionally, *TNNT2* mutations disrupt sarcomere-mitochondrial communication, activating the RAS/RAF1-p44/42 axis to promote mitochondrial fission (via MFF and DRP1 phosphorylation), ultimately driving DCM progression and fibrosis. (2) Metabolic-driven cardiomyopathies (DbCM, OIC, and CCM): diabetic cardiomyopathy (DbCM): Persistent hyperglycemia induces endothelial cell injury (EndMT) and activates the TGF-β/Smad and MAPK pathways. Elevated CaSR expression further triggers the Smurf2-ubiquitin proteasome system. Concurrently, the accumulation of advanced glycation end products (AGEs) activates RAGE-mediated NOX/ROS, ERK1/2, and NF-κB signaling, leading to cardiomyocyte apoptosis and extracellular matrix (ECM) deposition. Obesity-induced Cardiomyopathy (OIC): Hyperlipidemia-induced secretion of PDGF-D activates the PI3K/AKT pathway. Simultaneously, adipocyte-derived resisting binds to TLR-4, activating the JAK2/STAT3 axis to drive the transcription of profibrotic genes. Cirrhotic cardiomyopathy (CCM): Systemic metabolic defects stemming from hepatic dysfunction lead to elevated liver-derived TGF-β1. This systemic surge inhibits Notch1 signaling via DNA methylation-dependent mechanisms, exacerbating myocardial fibrosis.

Although the initial pathogenic causes of genetic-driven cardiomyopathies (HCM, DCM) and metabolic-driven cardiomyopathies (DbCM, OIC, CCM) are essentially different, their core downstream signaling pathways inducing MF are highly convergent. For instance, the classic TGF-β/Smad pathway serves as the central effector pathway throughout the progression^60^; the NF-κB inflammatory pathway initiates inflammatory cascades to participate in fibrotic progression^61^^,^^62^; the MAPK/ERK pathway plays a critical regulatory role in synergistically amplifying pathological remodeling and inflammatory responses^63^; additionally, the ROS/oxidative stress pathway acts as a general injury amplification mechanism.^60^^,^^61^ Meanwhile, MF in different subtypes of cardiomyopathy also exhibits distinct mechanistic features (Figure 3).

### Hypertrophic cardiomyopathy and myocardial fibrosis

HCM is characterized by left ventricular hypertrophy (LVH), myocardial hypercontractility, reduced compliance, myofibrillar disarray, and fibrosis.^64^ Some studies have shown that the incidence of MF is relatively high in patients with HCM.^64^ The presence of MF is associated with a 3.4-fold increased risk of MACE.^65^ Some studies have shown that among patients with HCM, those carrying sarcomere gene mutations exhibit more fibrosis than those without mutations.^66^

Mutations in the *MYBPC3* gene, which encodes cardiac myosin-binding protein C (cMyBP-C), are one of the most common etiologies of HCM.^67^ Recently, Zou et al. demonstrated that the downregulation of *MYBPC3* expression induced by this gene mutation first activates the NF-κB signaling pathway, enhancing its activity and inducing the release of inflammatory factors such as CCL2, IL-1β, IL-6, and TNF-α, thereby initiating the inflammatory response cascade. Subsequently, the activated NF-κB further upregulates the expression of TGF-β1, while promoting the activation of the HIF-1α signaling pathway and the enhancement of aerobic glycolysis. The synergistic effect of such signal regulation and metabolic remodeling drives the phenotypic transformation of cardiac fibroblasts into myofibroblasts, and simultaneously enhances the proliferation and migration capabilities of myofibroblasts while inhibiting their apoptosis.^62^ Mutations in the *MYH7* gene also represent a common etiology of HCM. Although the prevalence of *MYH7* mutations (20%–40%) in HCM is lower than that of *MYBPC3* mutations (40%–50%), one study indicates that the prevalence of MF is significantly greater in cases caused by the former.^68^ Studies illustrated that TGF-β/Smad2/3, ERK1/2 and Nox4/ROS pathways have synergistic effects on cardiac remodeling and inflammation in the *MYH7 R453C* mutation.^60^ Beyond the two primary genes, mutations in other genes (Table 1) may also be involved in the development and progression of MF in HCM.

**Table 1:** The key genes and a genetic polymorphism associated with myocardial fibrosis in HCM

Gene	Chromosomal location	Expressed protein	Model	Mechanism	Reference
*MYBPC3*	11p11	myosin-binding protein C 3	*MYBPC3*-R495Q knock-in pig model	NF-κB inflammation and TGF-β1/HIF-1α metabolic remodelling → myofibroblast activation	Zou et al.^62^
*MYH7*	14q12	β-myosin heavy chain 7	*MYH7*-R453C knock-in pig model	activates TGF-β/Smad, ERK, and Nox4/ROS pathways → inflammation and remodelling	Wang et al.^60^
*ACTC1*	15q14	actin, alpha cardiac muscle 1	*ACTC* E99K mouse model	increases Ca^2+^ sensitivity; fibrosis by 21 weeks	Song et al.^69^
*PLN*	6q22	phospholamban	cardiomyocyte model of HCM	altered Ca^2+^ buffering/handling → Ca^2+^-dependent signalling → cellular remodelling	Robinson et al.^70^
*AEBP1*	7p13	adipocyte enhancer-binding protein 1	human cardiac fibroblasts (*in vitro*)	negative regulator of TGF-β/Smad; loss-of-function promotes fibrotic activation	Liu et al.^71^
*PTN*	7q33	pleiotrophin	transverse aortic constriction mouse model	*PTN*-SDC4 pathway promotes fibroblast proliferation/migration	Sheng et al.^72^
*RETN* (−420C>G)	19p13.2	resisting	HCM patients	−420C>G increases resisting expression → myocardial fibrosis	Romero et al.^73^

Abbreviations: HCM, hypertrophic cardiomyopathy; NF-κB, nuclear factor kappa-B; TGF-β1, transforming growth factor beta 1; HIF-1α, hypoxia-inducible factor 1 alpha; Nox4, NADPH oxidase 4; ROS, reactive oxygen species; ERK, extracellular signal-regulated kinase; Ca^2+^, calcium ion; SDC4, syndecan 4.

### Dilated cardiomyopathy and myocardial fibrosis

DCM is a heterogeneous myocardial disease characterized by left ventricular dilatation and subsequent contractile dysfunction. Fibrosis plays an important role in the progression of DCM. In addition to some classic signaling pathways such as TGF-β and transition of fibroblasts to myofibroblasts, the mechanism at the genetic level is also key to connecting fibrosis and DCM.^74^ Wang et al. found that Bcl2-associated athanogene 3 (*BAG3*) in cardiac fibroblasts drives cardiac fibrosis by regulating TGF-β signaling in DCM.^75^ Antoine Muchir et al. found that *LMNA* mutation is a key pathogenic factor for DCM, and the abnormal Lamin A/C encoded by the mutation can activate the MAPK signaling pathway represented by ERK and JNK cascades, driving the progression of DCM.^63^ Subsequent studies further confirmed that MAPK inhibitors can effectively prevent MF in *LMNA* mutation-related DCM.^76^ Cardiac troponin T (cTnT) is encoded by the *TNNT2* gene and is essential for sarcomere assembly, contraction, and force generation in cardiomyocytes. A study by Ye et al. demonstrated that the cTnT p.K185E variant disrupts its interaction with 14-3-3 proteins, leading to the interruption of key molecular communication between sarcomeres and mitochondria, which in turn abnormally activates the RAS/RAF1-p44/42 signaling pathway. This promotes the phosphorylation of mitochondrial fission factor (MFF) and dynamin-related protein 1 (DRP1), enhancing aberrant mitochondrial fission and impairing mitochondrial function. These findings provide a new perspective on the pathogenesis of DCM caused by *TNNT2* sequence variations, and this pathological phenotype can be recapitulated with fibrotic features in three-dimensional cardiac organoid models.^77^ In addition, truncating variants in the *TTN* gene, which encodes the giant elastic protein titin, are the most common genetic subtype of DCM.^78^ However, its pathogenic mechanism remains debated. Current understanding suggests that disease onset likely results from multifactorial processes. Furthermore, *CFIRL*, a long noncoding RNA (lncRNA) significantly upregulated in DCM, has also been demonstrated to be associated with fibrosis.^79^ *MYH7,* associated with HCM also plays a definite role in DCM.^80^

### Diabetic cardiomyopathy and myocardial fibrosis

DbCM is defined as a cardiovascular complication specific to diabetes. It is characterized by structural remodeling of the myocardium, including hypertrophy and interstitial fibrosis, along with a progressive impairment of cardiac function mediated by hyperglycemia and its metabolic products.^81^ DbCM is often accompanied by hyperglycemia and insulin resistance, which promote the accumulation of advanced glycation end products (AGEs). These factors are typically associated with the development of MF.^82^ MF in DbCM is a multi-step, multicellular process that originates from cellular responses to oxidative stress, endoplasmic reticulum stress, and inflammation.^83^ In patients with diabetes, worse glycemic control was associated with a higher degree of MF. Therefore, tight glycemic control may prevent cardiac fibrosis in subjects with diabetes.

Persistent hyperglycemia can directly lead to significant activation of cardiac fibroblasts, promoting their differentiation into myofibroblasts. This process subsequently results in ECM accumulation and MF.^84^ *In vitro* studies have demonstrated that HG increases the expression and activity of TGF-β.^85^ TGF-β mainly activates the typical Smad2/3 and MAPK signaling pathways, promoting the synthesis and secretion of various ECM components by cardiac fibroblasts, such as collagen types I and III. Cardiovascular complications in diabetes are significantly driven by endothelial dysfunction. Diabetic endothelial injury can trigger endothelial-to-mesenchymal transition (EndMT), promoting the phenotypic conversion of endothelial cells into myofibroblasts. This process provides an additional source of activated fibroblasts, thereby contributing to the progression of MF. Sustained hyperglycemia stimulation also upregulates the expression of the calcium-sensing receptor (CaSR) in cardiac fibroblasts. Increased CaSR expression activates the Smurf2-ubiquitin proteasome pathway and autophagy, leading to excessive cardiac fibroblast proliferation and substantial collagen deposition, which in turn induces MF.^86^ Recent studies have demonstrated that PDZK1 expression is downregulated in the cardiac tissue of a DbCM mouse model, which activates cardiac fibroblasts via EGFR phosphorylation and PI3K/AKT signaling, ultimately leading to cardiac fibrosis. These findings identify PDZK1 as a key regulator of MF in DbCM, underscoring its potential as a therapeutic target for anti-fibrotic interventions.^87^

Cardiomyocytes play a critical role in the pathogenesis of MF associated with DbCM. Prolonged dysregulation of glucose metabolism can induce cardiomyocyte death. Insulin resistance reduces cardiac glucose uptake by impairing the translocation of glucose transporter 4 (GLUT4) to the cell membrane.^82^ Concurrently, the increased fatty acid uptake regulated by fatty acid translocase promotes fatty acid accumulation and activates Peroxisome Proliferator-Activated Receptors in diabetes,^88^ thereby shifting the metabolic preference of cardiomyocytes toward fatty acid β-oxidation. This metabolic switch elevates cellular metabolic stress, ultimately leading to cardiomyocyte death.^89^ Furthermore, the upregulation of endoplasmic reticulum stress (ERS) and ROS in DbCM are key contributors to cardiomyocyte apoptosis,^90^ which can ultimately compromise cardiac function and initiate MF.^91^

Besides, the rate of AGEs accumulation is significantly accelerated in diabetic conditions.^92^ Studies demonstrate that AGEs directly upregulate both the gene expression and protein synthesis of type I collagen in adult rat cardiac fibroblasts.^93^ The binding of AGEs to their receptor RAGE activates multiple signaling cascades, including the NADPH oxidase/reactive oxygen species (NOX/ROS), ERK1/2, and NF-κB pathways.^61^ This activation drives downstream pathological events such as enhanced oxidative stress, ECM remodeling, and myofibroblast differentiation. Furthermore, elevated AGEs within diabetic cardiac collagen enhance cardiac fibroblast contractility via RAGE-mediated signaling.^92^ This increased myofibroblast differentiation may potentiate matrix contraction,^94^ which in turn reinforces myofibroblast trans differentiation, thereby establishing a profibrotic positive-feedback loop.^92^

### Obesity-induced cardiomyopathy and myocardial fibrosis

OIC is a secondary myocardial lesion caused by chronic obesity and lipid metabolic disorders, characterized by chronic inflammation-mediated MF, cardiac hypertrophy, and cardiac dysfunction.^95^ Obesity is often accompanied by chronic low-grade inflammation, which may contribute to the development, progression, and remodeling associated with MF.^96^ Obesity is often associated with hyperlipidemia and elevated levels of resisting.

Hyperlipidemia can promote fibrosis through immune cells. Studies have found that disordered lipid metabolism may lead to increased secretion of full-length platelet-derived growth factor-D (PDGF-D) from adipose tissue into the circulation. Urokinase plasminogen activator (uPA), produced by activated macrophages in myocardial tissue, then cleaves circulating PDGF-D into its active form. This active PDGF-D subsequently activates the PI3K/AKT signaling pathway in cardiac fibroblasts, thereby promoting fibrosis.^97^ In addition, resisting plays a pivotal role in the pathogenesis of MF driven by hyperlipidemia associated with disordered glucose metabolism. Resisting, an adipocyte-derived hormone, is closely linked to obesity, insulin resistance, and diabetes.^98^ Studies demonstrate that cardiac-specific overexpression of resisting induces a profibrotic phenotype characterized by elevated expression of key fibroblast markers and ECM proteins, including α-SMA, collagen type I alpha 1 chain, connective tissue growth factor, fibronectin, lysyl oxidase, and splicing factor 2.^99^ Mechanistically, resisting binding to Toll-like receptor 4 activates Janus kinase 2, leading to the phosphorylation of signal transducer and activator of transcription 3 (STAT3). Phosphorylated STAT3 translocate into the nucleus and promotes the transcription of profibrotic genes such as type I collagen and fibronectin 1, ultimately stimulating cardiac fibroblast proliferation and MF.^100^

### Cirrhotic cardiomyopathy and myocardial fibrosis

CCM is a myocardial dysfunction secondary to hepatic insufficiency in liver cirrhosis, the pathogenesis of which is closely associated with systemic metabolic defects caused by hepatic dysfunction.^56^ CCM is associated with myocardial hypertrophy (MH) and subendocardial edema (SE), and can progress to fibrosis.^101^ The early stage of MF is diffuse and potentially reversible, whereas the more advanced subendocardial segmental fibrosis is considered permanent.^101^ Cardiac fibrosis stands as the most common pathological feature of CCM, which can induce systemic inflammation and impair the functional integrity of other organs.^102^ Latest research by Sun et al. indicates that in a cirrhotic mouse model established using the classic inducer carbon tetrachloride (CCl_4_), the level of TGF-β1 in the circulatory system is elevated, which is consistent with the findings observed in human patients with CCM. Notably, studies have confirmed that the elevated TGF-β1 is mainly derived from the liver. The increased TGF-β1 inhibits Notch1 through a DNA methylation-dependent mechanism, thereby exacerbating the progression of cardiac fibrosis.^102^

## Hypertension and myocardial fibrosis

Hypertension, characterized by persistently elevated systemic arterial pressure, represents a highly prevalent chronic condition and a major modifiable risk factor for global cardiovascular morbidity and mortality.^103^ Long-term elevated blood pressure, as seen in hypertension, will increase the cardiac load and lead to MF.

The inappropriate activation of RAAS constitutes a crucial pathogenic factor in the development and progression of hypertension. Through the synergistic activation of downstream pathways by the major RAAS effector molecules Ang II and aldosterone, pathological ECM remodeling is induced, ultimately resulting in irreversible MF. In addition, mechanical stress that occurs due to hypertension-induced left ventricular pressure overload can be sensed by resident cardiac fibroblast mechanosensitive receptors that mediate mechanotransduction signaling involved in fibroblast activation, as well as differentiation into myofibroblasts.^104^ In addition to the aforementioned mechanisms by which increased mechanical pressure induces MF, Yan et al. found that the levels of Wnt5a and Wnt11 are increased in the serum of patients with hypertension. Pressure overload enhances the secretion of Wnt5a or Wnt11 from cardiomyocytes and cardiac fibroblasts. *In vitro* experiments have shown that exogenous Wnt5a or Wnt11 exerts pro-fibrotic effects on cardiac fibroblasts by activating the ERK and p38 (fibrosis-related signaling) pathways, promoting EGFR phosphorylation, and increasing the expression of Frizzled 5 (FZD5).^105^

## Mechanism comparison and treatment prospects of myocardial fibrosis

MF represents a common final pathway for various cardiac diseases; however, the initiating factors and core mechanisms driving this pathological process differ significantly among distinct conditions. Table 2 aims to systematically delineate and compare the similarities and differences in the triggers and core mechanisms of fibrosis in different cardiovascular diseases, thereby providing a clear framework for a deeper understanding of their pathophysiology and for exploring targeted therapeutic strategies.

**Table 2:** Comparison of triggering factors and mechanisms of myocardial fibrosis across different diseases

Disease	Triggering factors	Unique mechanisms
Myocardial infarction (MI)	acute coronary occlusion → ischemia/necrosis → DAMP release	• PANX1/P2rx7/AREG • VSIG4^+^/TGF-β expression • NAT10-mediated RNA ac4C acetylation
Heart failure (HF)	oxidative stress/mechanical stress	• pressure overload activates mechanotransduction pathways • volume overload leads to dysregulated ECM degradation • VSIG4 regulates fibrosis via immune/inflammatory responses • SNO-PKM2 enhances fibroblast activation by promoting mitochondrial dysfunction • CircIGF1R regulates fibroblast proliferation by inhibiting glycolysis
Hypertrophic cardiomyopathy (HCM)	sarcomere gene mutations (*MYH7, MYBPC3*) → myocyte disarray/hypertrophy → ischemia and cellular stress	• NF-κB-driven inflammation and TGF-β1/HIF-1α-mediated metabolic remodelling • concurrent activation of TGF-β/Smad2/3, ERK1/2, and Nox4/ROS pathways
Dilated cardiomyopathy (DCM)	genetic factors (*TTN, LMNA*)/acquired injury → ECM exposure and integrin signalling	• *LMNA* mutations activate MAPK (ERK/JNK) cascades • *TNNT2* mutations disrupt sarcomere-mitochondrial communication via RAS/RAF1-p44/42 axis • *BAG3* in cardiac fibroblasts drives fibrosis by regulating TGF-β signalling
Diabetic cardiomyopathy (DbCM)	hyperglycaemia/lipotoxicity → ROS and AGEs → RAGE activation → metabolic remodelling	• AGEs serve as disease-specific initiating factors • hyperglycaemia-induced End MT provides additional fibroblast source • CaSR upregulation activates Smurf2-ubiquitin proteasome pathway • PDZK1 downregulation activates EGFR/PI3K/AKT signalling
Obesity-induced cardiomyopathy (OIC)	chronic obesity → lipid disorders and inflammation → immune activation	• PDGF-D/uPA from adipose-macrophage axis activates PI3K/AKT pathway • resisting binds TLR4, activating JAK2/STAT3 to drive pro-fibrotic gene transcription
Cirrhotic cardiomyopathy (CCM)	decreased hepatic clearance	• liver-derived TGF-β1 exacerbates cardiac fibrosis by inhibiting Notch1 via DNA methylation-dependent mechanism
Hypertension	persistently elevated blood pressure	• aberrant RAAS activation directly disrupts ECM balance • pressure overload enhances Wnt5a/Wnt11 secretion, promoting fibrosis via FZD5-EGFR crosstalk

DAMPs, damage-associated molecular patterns; PANX1, pannexin 1; VSIG4, V-set and immunoglobulin domain-containing 4; NAT10, N-acetyltransferase 10; ECM, extracellular matrix; SNO-PKM2, S-nitrosylated pyruvate kinase M2; CircIGF1R, circular RNA IGF1R; NF-κB, nuclear factor kappa-B; HIF-1α, hypoxia-inducible factor 1 alpha; Nox4, NADPH oxidase 4; ERK, extracellular signal-regulated kinase; JNK, c-Jun N-terminal kinase; MAPK, mitogen-activated protein kinase; BAG3, Bcl2-associated athanogene 3; EndMT, endothelial-to-mesenchymal transition; AGEs, advanced glycation end products; RAGE, receptor for AGEs; CaSR, calcium-sensing receptor; PDZK1, PDZ domain containing 1; EGFR, epidermal growth factor receptor; PI3K, phosphoinositide 3-kinase; AKT, protein kinase B; PDGF-D, platelet-derived growth factor D; uPA, urokinase plasminogen activator; TLR4, Toll-like receptor 4; JAK2, Janus kinase 2; STAT3, signal transducer and activator of transcription 3; RAAS, renin-angiotensin-aldosterone system; FZD5, Frizzled 5; TGF-β, transforming growth factor beta; ROS, reactive oxygen species.

Based on the above mechanisms, many clinical studies have shown that some drugs have anti-MF effects. Abnormal activation of the TGF-β/Smad signaling pathway is the core mechanism driving the occurrence and progression of MF. Pirfenidone (5-methyl-1-phenyl-2-[1H]-pyridine) is an oral antifibrotic agent that exerts antifibrotic effects by downregulating the levels of TGF-β and proinflammatory cytokines.^106^ Cardiac magnetic resonance (CMR) assesses the extent of myocardial interstitial fibrosis via changes in extracellular volume (ECV). A phase II, double-blind clinical trial by Gavin A. Lewis et al. in patients with HF demonstrated that, compared with placebo, 52 weeks of pirfenidone treatment significantly reduced myocardial ECV in patients with HFpEF and MF (between-group difference, −1.21%; 95% CI, −2.12 to −0.31; *p* = 0.009).^107^ Sodium-glucose cotransporter 2 inhibitors (SGLT2i) are a class of drugs that primarily target sodium-glucose cotransporter 2 (SGLT2) in the S1/S2 segments of the renal proximal tubule and reduce the risk of MACE.^108^ These drugs exert anti-MF effects by regulating multiple signaling pathways, including the TGF-β1/Smad2/3 pathway, the MAPK pathway, and the AMPK/mTOR pathway.^109^ The DAPA-EAT trial by Akira Taruya et al. in patients with HF demonstrated that compared with standard therapy alone (non-SGLT2i group), dapagliflozin intervention significantly reduced left ventricular MF volume (between-group difference, −5.32; 95%CI, −8.00 to −2.63; *p* = 0.009).^110^ A randomized open-label controlled trial by Hongmei Yan et al. in participants at high risk of type 2 diabetes mellitus showed that, compared with the sitagliptin group, canagliflozin significantly reduced myocardial ECV levels (adjusted mean difference [AMD]: −3.67%; 95%CI −5.33 to −2.01; *p* < 0.001).^111^

Furthermore, RAAS inhibitors (RAASi) exert their effects by targeting different sites of the system, and mainly include renin inhibitors, ACE inhibitors (ACEi), angiotensin II type 1 receptor blockers (ARBs), and mineralocorticoid receptor antagonists (MRAs). Eplerenone is a steroidal agent among MRAs. A randomized controlled trial by Stavroula Papapostolou et al. in patients with non-obstructive HCM demonstrated that 12 months of eplerenone treatment significantly improved diffuse MF compared with the placebo group, as assessed by CMR imaging.^112^ Finer none is the first and only nonsteroidal MRA approved by the FDA for the treatment of HF with mildly reduced ejection fraction (HFmrEF). The FINEARTS-HF trial by Riccardo M. Inciardi et al. demonstrated that, compared with placebo finer none reduced the rate of rehospitalization in patients with HF.^113^ Furthermore, based on existing research from trials such as FINEARTS-HF and the results of ongoing studies, Muhammad Furqan et al. proposed that finer none may help reverse cardiac and vascular remodeling, thereby improving cardiac function. Sacubitril/valsartan is the first angiotensin receptor-neprilysin inhibitor (ARNI). It is beneficial for multiple events associated with cardiac remodeling.^114^ A REVERSE-LVH randomized phase 2 trial by Vivian Lee et al. in patients with essential hypertension and LVH found that sacubitril/valsartan significantly promoted the regression of diffuse myocardial interstitial fibrosis compared with valsartan alone.^115^

In addition, there are also some emerging treatment methods. Vericiguat is a novel soluble guanylate cyclase (sGC) stimulator. The VICTORIA trial by Paul W Armstrong et al. in patients with HFrEF demonstrated that vericiguat can prevent and even reverse left ventricular MF.^116^ Its potential mechanism is to upregulate the cGMP-PKG signaling pathway, inhibit oxidative stress and myocardial apoptosis, and thus improve cardiac remodeling and systolic function.^117^ Recently, 2-APQC, a targeted SIRT3 activator, has been reported to reduce fibrosis by regulating mitochondrial homeostasis. Fibroblast activation protein (FAP) receptor is expressed by cardiac myofibroblasts. A novel approach utilizing *in vivo* engineered chimeric antigen receptor (CAR) T cells via lipid nanoparticles encapsulating mRNA encoding anti-FAP receptor CARs can specifically deplete activated fibroblasts, thereby alleviating MF.^118^

All of these novel drugs exhibit promise as additions to the therapeutic arsenal against MF, but further evidence is needed in the future. Also, targeting key factors of the signaling pathway, as well as regulating the crucial enzymes of energy metabolism and epigenetic modifications, holds the potential as novel targeted therapies for MF in the future. The latest research has shown us another aspect of fibrosis treatment. The human induced pluripotent stem cell (iPSC) derived model revealed new driving factors for fibrosis.^119^ In addition, the development of significantly specific fibroblast markers and multiple simultaneous analyses of genomic variants, transcriptional networks, protein-protein interactions, and metabolic characteristics reveal that the treatment of fibrosis in the future will develop toward precision medicine treatment.^120^

## Conclusion

This review synthesizes evidence demonstrating that although diverse diseases originate from distinct triggers, they ultimately converge upon a shared downstream fibrotic process driven by core profibrotic signaling pathways—including TGF-β/Smad, MAPK, and PI3K/Akt. Research on the mechanisms of MF in the context of different cardiovascular diseases is of great significance for achieving precise clinical treatment. However, Current clinical practice still lacks two key elements: early diagnostic biomarkers and broadly effective anti-fibrotic drugs. Future research should focus on identifying disease-specific and stage-specific biomarkers for early detection, developing targeted therapeutic strategies that address the initiating mechanisms, and validating combination therapies that simultaneously modulate inflammation, metabolism, and fibroblast activation. This will accelerate the transformation of MF from an irreversible endpoint to a clinically manageable goal.

## Acknowledgments

This work was supported by 10.13039/501100004480Shanxi Natural Science Foundation (202403021221298).

## Author contributions

Conceptualization, H.C.; writing – original draft, B.Z., X.C., and H.L.; writing – review and editing, H.C., B.Z., X.C., S.F., J.P., H.L., H.Y., and W.L.; funding acquisition, H.C.; supervision, H.C.

## Declaration of interests

The authors declare no competing interests.

## Declaration of generative AI and AI-assisted technologies in the writing process

The authors did not use generative AI or AI-assisted technologies in the development of this manuscript.
